# Evaluation of the Call for a Kit intervention to increase bowel cancer screening uptake in Lancashire, England

**DOI:** 10.1177/09691413221089184

**Published:** 2022-04-12

**Authors:** Sandro T Stoffel, Lesley McGregor, Yasemin Hirst, Sahida Hanif, Lorraine Morris, Christian von Wagner

**Affiliations:** 1 Research Department of Behavioural Science and Health, University College London, London, UK; 2 Institute for Pharmaceutical Medicine, 27209 University of Basel, Basel, Switzerland; 3 Division of Psychology, Faculty of Natural Sciences, 7622 University of Stirling, Stirling, UK; 4 1756 Blackpool Teaching Hospitals, NHS Foundation Trust, Blackpool, UK

**Keywords:** Cancer screening, health promotion, faecal occult blood test, Black and minority ethnic groups

## Abstract

**Objective:**

To evaluate the ‘Call for a Kit’ health promotion intervention that was initiated in Lancashire, England to improve bowel cancer screening uptake.

**Methods:**

Within the intervention, screening non-responders are called and invited to attend a consultation with a health promotion team member at their primary care practice. In this audit, we analysed the proportion of those contacted who attended the in-person clinic versus those who received a phone consultation, the number returning a test kit from in-person versus phone consultations, and the extent to which test kit return was moderated by sociodemographic characteristics.

**Results:**

In 2019, 68 practices participated in the intervention which led to 10,772 individuals being contacted; 2464 accepted the invitation to an in-person consultation, of whom 1943 attended. A further 1065 agreed to and attended a consultation over the phone. The 3008 consultations resulted in 2890 test kits being ordered, of which 1608 (55.6%) were returned. The intervention therefore yielded a 14.9% response rate in the total cohort; 71.5% of test kits came from individuals attending the in-person consultation. Women and those registered with a practice in socioeconomically deprived areas were less likely to return the test kit. Individuals with a black, mixed or a non-Indian/Pakistani Asian ethnic background were significantly more likely to accept the offer of an in-person consultation and return the test kit.

**Conclusion:**

Our analysis demonstrated the strong likelihood of people returning a test kit after an in-person appointment but also the usefulness of using phone consultations as a safety net for people unable or unwilling to attend in-person clinics.

## Introduction

Colorectal cancer (CRC) is one of three cancer types (alongside breast and cervical cancer) that can be reliably detected at an asymptomatic stage, when treatment is associated with significantly better clinical outcomes.^1^ However, the greatest proportion of CRCs are diagnosed symptomatically. Moreover, many of these cancers are diagnosed after an emergency presentation, when the cancer has already progressed and the chance of 5-year survival is at its lowest^2^ In the UK, one way to increase the proportion of CRC cases diagnosed early via the screening programme has been to promote uptake of the National Bowel Cancer Screening Programme (BCSP). The BCSP was rolled out in 2006. The programme initially offered a guaiac faecal occult blood test (FOBt) which was completed at home by providing three small stool samples from separate bowel motions. Uptake of the test was low and socially graded. During the initial roll-out of the English Bowel (Colorectal) Cancer Screening programme, between 2006 and 2009, uptake was 54% ranging from 35% in the most to 61% in the least socioeconomically deprived areas in England.^3^ The most ethnically diverse areas also had lower uptake (38%) than other areas. Over the subsequent 6 years, uptake continued to be socially graded between the most and the least deprived area-level socio-economic deprivation quintiles (43% vs. 57%) and the most and the least area-based ethnic diversity quintiles (41% vs. 56%) as well as between men and women (47% vs. 56%).^4^ Multivariate analysis confirmed independent effects of year, deprivation, ethnicity and gender on uptake.

In 2019, the test was replaced by a single sample stool test (the faecal immunochemical test; FIT). A pilot study found that uptake of this test is higher (7.1%), particularly among men and previous non-responders, but that socioeconomic and ethnic disparities persist^5^ Research into the reasons for these inequalities identified limitations posed by written English; reliance on younger family members; low awareness of colorectal cancer and screening; and difficulties associated with faeces.^6^

There are many techniques or interventions which have been associated with small to moderate improvements in uptake, including a combination of various different forms of general practitioner (GP) endorsement and reminder calls and letters.^7,8^ In Lancashire, an ethnically diverse county in North West England, the National Health Service (NHS) initiated an intervention named “Call for A Kit” (CFAK) in 2015, which aimed to improve CRC screening uptake.^9^ The health promotion team for the area encouraged local GP practices to participate in CFAK by delivering in-person consultations to individuals identified as not being up to date with CRC screening. The health promotion team has been able to demonstrate that the interventions works in principle through a process evaluation and by looking at top-line data on FOBt/FIT participation by GP practice. The aim of this audit was to offer a more in-depth analysis that would take account of each of the steps involved in delivering the intervention including: 1) the reach of the intervention, i.e., the proportion of previous non-responders who were successfully contacted; 2) the proportion who attended the in-person clinic versus those who received a phone consultation because they were unable or unwilling to attend the clinic; 3) the number returning a test kit from in-person versus phone consultations; and 4) the extent to which test kit return was moderated by sociodemographic characteristics.

## Methods

We used data from 68 GP practices in Lancashire, who participated in CFAK. The local health promotion team consists of 5 individuals: 1 has an NHS clinical nursing/public health background and the other 4 have voluntary sector community engagement backgrounds. All the team are skilled and experienced community development workers. The intervention was developed and implemented as a pilot in 2017. Due to the success of the pilot the intervention was expanded into a project across Lancashire with 4 additional team members. The team learnt and further developed the model whilst delivering the project.

In 2019, this team called up individuals registered with the practice who did not return their test kit within 13 weeks after receiving their most recent CRC screening invitation. Each individual’s CRC screening episode closes at 12 weeks after the initial invitation was sent out and non-responder status is established by week 13. During the initial call, individuals were invited to a 15-min in-person consultation at their GP practice.

### In-person consultation

The in-person meeting was held in the person’s preferred language and, where possible, with a same sex health promotion specialist During the consultation, the health promotion specialist showed participants the screening kit, and discussed the barriers to taking part in CRC screening. The session also included a video presentation of how the test kit is completed and the offer of a replacement test kit (which, if the person consented, was sent to their home via the North West and Midlands Screening Hub). Additionally, individuals were given additional information from the BCSP website together with the number of the helpline.

### Phone-based consultations

Individuals who were not able or refused to attend an in-person consultation were given the opportunity to continue with the call and receive a brief phone-based consultation. During the call, non-responders were invited to discuss their barriers to completing the test kit, and given the opportunity to request a replacement test kit.

### Demographic variables

We used the postcode of the GP practice to derive the socio-economic deprivation score for the practice using the Index of Multiple Deprivation.^10^ We had the postcodes of all the practices, therefore there are no missing data. Gender and ethnicity were collected from the individual at the in-person clinic appointment or during a phone consultation.

### Test kit return

Test kit return was checked by GP practices 13 weeks after issue of the replacement test kit in line with the duration of a BCSP screening episode.

### Statistical analysis

Individuals’ gender and ethnicity (White, Indian, Pakistani, or Black, mixed or other ethnicities), and the area deprivation score (Index of Multiple Deprivation; IMD) of the GP practice were used for the statistical analysis of the CFAK intervention.^10^

We used multivariate logistic regressions to analyse the return of test kits and acceptance of the consultations according to an intention-to-treat and a per-protocol basis. In contrast to the intention-to-treat analysis, the per-protocol analysis excluded individuals who could not be reached by phone or who declined the invitation for the consultations. All statistical analysis was conducted using Stata/IC version 16.0 (StataCorp LP, College Station, TX, USA).

## Results

**Figure 1.**
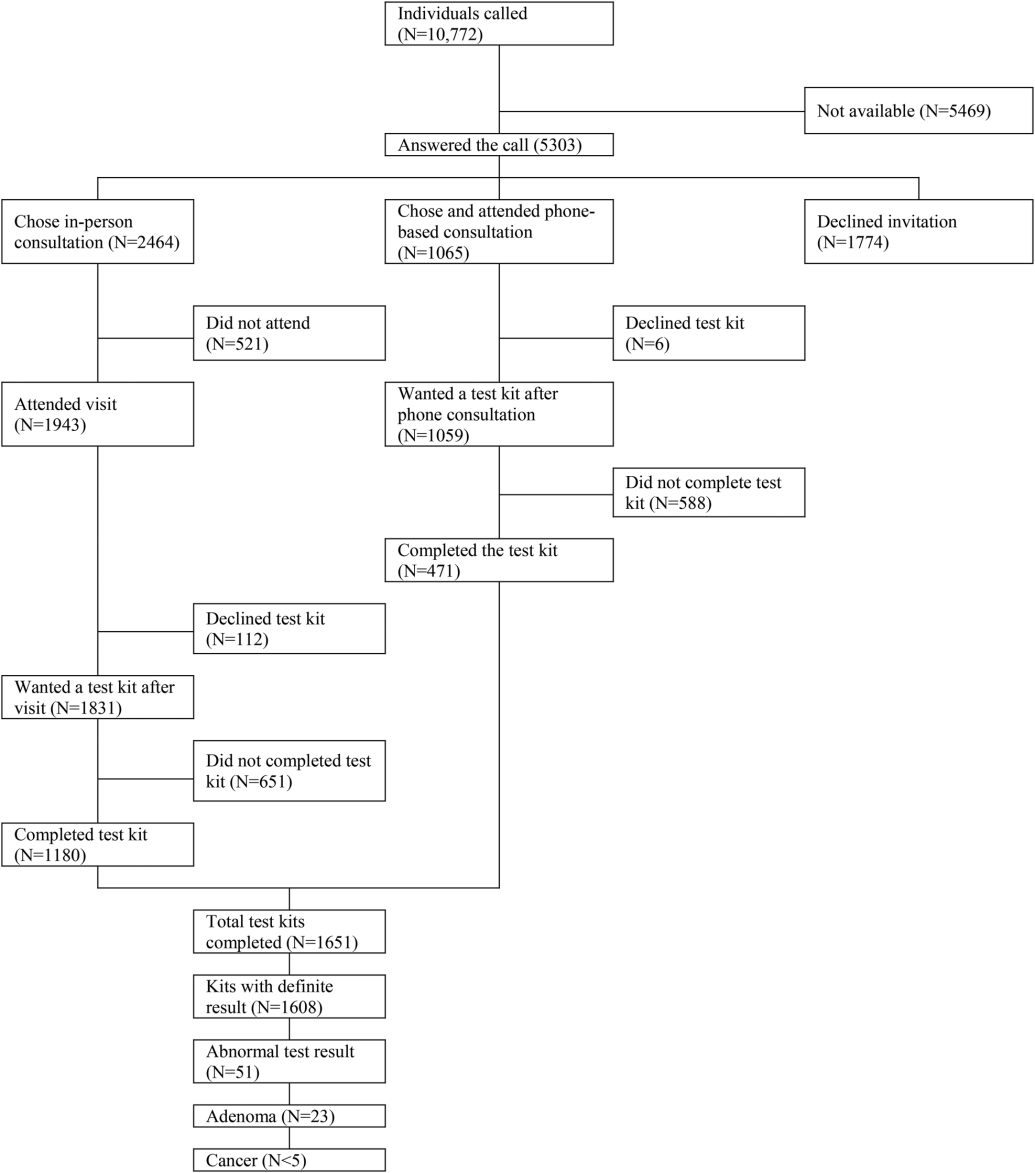
Flow chart describing the study participants from 68 GP practices in 2019.

Figure 1 shows that out of the 10,772 non-responders called, 5303 (49.2%) answered the call. Of these, 3529 (66.5%) accepted the invitation for a consultation: 2464/3529 (69.8%) accepted the offer of an in-person consultation while 1059/3529 (30.2%) were either unable or unwilling to attend an in-person meeting and chose the offer of the phone-based consultation (see Table S1 in the online supplementary file for the characteristics of the study sample and Table S2 for the differences in demographics between those who accepted the face-to-face meeting and those who chose the phone consultation). In the case of in-person consultations, 521/2464 (21.1%) did not attend the meeting. In total, 3008 individuals had a consultation. Of these, 1943 (64.6%) took place in person and 1065 (35.4%) over the phone. Almost all of the recipients of the intervention (N = 2890/3008; 96.1%) requested a new test kit of which 1651 were returned (57.1%). Most test kits came from individuals attending the in-person consultation (1180/1651, 71.5%). Phone-based consultations contributed an additional 471 completed test kits (471/1651, 28.5%; see Figure 1).

## A multivariable model of receiving the phone or in-person consultation

Table 1 shows that individuals of Pakistani and black mixed background were more likely to attend an in-person or phone-based consultation (133/281, 47.3% and 55/88, 61.8% respectively) compared with people from a white ethnic background (3218/10,035, 32.1%; adjusted odds ratio (OR) 1.80, 95% confidence interval (CI) 1.41–2.30 and adjusted OR 3.41, 95% CI 2.21–5.25 respectively). The adjusted regression shows that being registered at a GP practice in a less socioeconomically deprived area was positively associated with accepting either an in-person or phone-based consultation.

**Table 1.** Accepting invitation for consultation (*N*  =  10,772).

		Accepting invitation for either in-person or phone-based consultation					
				Unadjusted model		Adjusted model	
	N	N	(%)	OR	95% CI	OR	95% CI
Overall	10,772	3529	(32.8)				
Gender							
Female	4643	1469	(31.3)	Ref.		Ref.	
Male	6129	2060	(33.6)	1.096	1.008–1.187*	1.084	0.998–1.177
Ethnicity							
White	10,035	3218	(32.1)	Ref.		Ref.	
Indian	367	123	(33.5)	1.068	0.856–1.332	0.961	0.767–1.204
Pakistani	281	133	(47.3)	1.904	1.501–2.415**	1.802	1.413–2.298**
Black, mixed and others	89	55	(61.8)	3.427	2.230–5.266**	3.408	2.212–5.250**
Area level deprivation							
IMD quintile 5 [3–1529]	2397	767	(28.7)	Ref.		Ref.	
IMD quintile 4 [1542–3369]	2414	479	(33.9)	1.277	1.112–1.467**	1.205	1.046–1.388**
IMD quintile 3 [3396–6415]	1871	590	(31.4)	1.139	1.002–1.295*	1.140	1.002–1.298*
IMD quintile 2 [6524–11,645]	1417	896	(37.1)	1.467	1.304–1.650**	1.428	1.267–1.610**
IMD quintile 1 [11,685–26,146]	2673	797	(33.2)	1.238	1.099–1.395**	1.257	1.115–1.417**

* *p* < 0.05; ** *p* < 0.01.

CI: confidence interval; IMD: index of multiple deprivation; OR: odds ratio.

Note: IMD quintiles are calculated based on the rank of the GP practice which ranges from 1 (the most deprived area) to 32,844 (the least deprived area).

## Multivariable model of FIT return

### Intention-to-treat analysis

Investigating the return of the test kit based on the total number of calls made revealed several associations (see Table S3 for the characteristics of those who returned the test kit). Test kit return was associated with deprivation. Individuals whose GP practice was situated in a less deprived area were more likely to complete their test kit compared with those registered with practices in more deprived areas (see Table 2). Men were also more likely to return the kit than women (16.0% vs. 14.4%; adjusted OR 1.13, 95% CI 1.01–1.26). Individuals with a black, mixed or ‘other’ ethnic background were also more likely to complete the kit than those with a white background (33.7% vs. 15.0%; adjusted OR 2.97, 95% CI 1.90–4.65).

**Table 2.** Returning the test kit (intention-to-treat analysis, N  =  10,772).

		Returning the test kit					
				Unadjusted model		Adjusted model	
	N	N	(%)	OR	95% CI	OR	95% CI
Overall	10,772	1651	(15.3)				
Gender							
Female	4643	669	(14.4)	Ref.		Ref.	
Male	6129	982	(16.0)	1.133	1.019–1.261*	1.129	1.013–1.257*
Ethnicity							
White	10,035	1510	(15.0)	Ref.		Ref.	
Indian	367	58	(15.8)	1.060	0.796–1.410	0.954	0.713–1.276
Pakistani	281	53	(18.9)	1.312	0.968–1.778	1.241	0.910–1.693
Black, mixed and others	89	30	(33.7)	2.871	1.843–4.470**	2.972	1.900–4.648**
Area level deprivation							
IMD quintile 5 [3–1529]	2397	313	(11.7)	Ref.		Ref.	
IMD quintile 4 [1542–3369]	2414	225	(15.9)	1.430	1.189–1.712**	1.292	1.154–1.680**
IMD quintile 3 [3396–6415]	1871	264	(14.1)	1.234	1.035–1.471*	1.249	1.047–1.490*
IMD quintile 2 [6524–11,645]	1417	430	(17.8)	1.634	1.396–1.913**	1.606	1.369–1.885**
IMD quintile 1 [11,685–26,146]	2673	419	(17.5)	1.597	1.364–1.871**	1.620	1.383–1.898**
N						10,772	

* *p* < 0.05; ** *p* < 0.01.

CI: confidence interval; IMD: index of multiple deprivation; OR: odds ratio.

Note: IMD quintiles are calculated based on the rank of the GP practice which ranges from 1 (the most deprived area) to 32,844 (the least deprived area).

### Per protocol analysis

Of the 3008 men and women who received an in-person or phone-based consultation, 1651 (54.9%) completed and returned the test kit (see Table S4). Test kit return was associated with deprivation. Individuals whose GP practice was situated in a less deprived area were more likely to complete their test kit compared with those registered with practices in more deprived areas (61.8%, adjusted OR 1.56, 95% CI 1.25–1.93 vs. 47.3%, adjusted OR 1.79, 95% CI 1.44–2.23). There was no association between test kit return with gender or ethnicity.

## Discussion

The use of in-person or phone-based consultations with previous non-responders has been found to be highly effective. Despite unsuccessful attempts to reach previous non-responders by phone, 1 in 7 of those individuals went on to return an adequate test kit. While the aim of the clinics was to provide in-person support for non-responders it is worth noting that 28.5% of test kits returned came after a phone-based consultation, emphasizing the importance of providing this format as an additional safety net for those unable or unwilling to attend their GP practice.

While the CFAK intervention appears to be more effective for individuals who are registered with GPs situated in less deprived areas, there was no evidence of individuals from ethnic minority backgrounds being less likely to respond. On the contrary, individuals of black, mixed or non-Indian/Pakistani Asian ethnic background were more likely to respond to the invitation to attend a CFAK clinic. However, we should note that this group was small and heterogeneous, and more data are needed to understand which ethnic groups are particularly likely to benefit from CFAK interventions.

Our audit was limited in a number of ways, including only having access to data on area deprivation for the GP practice the patient was registered with. Furthermore, no data on the in-person and phone-call consultations was recorded; however, observations from the health promotion team suggest that consultations were consistent and similar in content. In patient navigation interventions, patients are helped to identify and circumvent individual barriers to healthcare, which is essentially what CFAK aimed to do.^11^ Future research should monitor the content of conversations more closely to better understand which barriers were most prominent and which solutions were or could be the most successful. This may then inform the delivery of initial invitations and increase prompt participation in the BCSP. More research is needed to optimise initial contact (e.g. by identifying better times to call or leaving voicemails).

At the time at which people in this study were invited, the programme was still using the guaiac FOBt. Future audits will be able to highlight how the effectiveness of this intervention is affected by a more user-friendly test Finally, the observation that a large proportion of individuals invited ended up having phone-based consultations raises the question of the comparative effectiveness of phone versus in-person consultations. Given the greater cost of running in-person clinics for the health service and the opportunity costs for those invited, it would be useful to directly compare both offers (using randomisation) and conduct a formal cost-effectiveness analysis. This is particularly pertinent in light of potential restrictions to physically accessing GP practices during the ongoing COVID-19 pandemic.^12,13^

In conclusion, this study shows that the CFAK intervention was successful in getting 15.3% of the non-attenders targeted to participate in the CRC screening programme. The in-person and phone-based consultations were effective in motivating more than half of the participants into ordering and returning a test kit.

## Supplemental Material

sj-docx-1-msc-10.1177_09691413221089184 - Supplemental material for Evaluation of the Call for a Kit intervention to increase bowel cancer screening uptake in Lancashire, England Click here for additional data file. Supplemental material, sj-docx-1-msc-10.1177_09691413221089184 for Evaluation of the Call for a Kit intervention to increase bowel cancer screening uptake in Lancashire, England by Sandro T Stoffel, Lesley McGregor, Yasemin Hirst, Sahida Hanif, Lorraine Morris and Christian von Wagner in Journal of Medical Screening

## References

[1] Logan RF Patnick J Nickerson C , et al. Outcomes of the Bowel Cancer Screening Programme (BCSP) in England after the first 1 million tests. Gut 2012; 61: 1439–1446. 22156981 10.1136/gutjnl-2011-300843 PMC3437782

[2] Cancer Research UK. Survival. https://www.cancerresearchuk.org/about-cancer/bowel-cancer/survival (2020, accessed 18 June 2021).

[3] Von Wagner C Baio G Raine R , et al. Inequalities in participation in an organized national colorectal cancer screening programme: results from the first 2.6 million invitations in England. Int J Epidemiol 2011; 40: 712–718. 21330344 10.1093/ije/dyr008

[4] Hirst Y Stoffel S Baio G , et al. Uptake of the English Bowel (Colorectal) Cancer Screening Programme: an update 5 years after the full roll-out. Eur J Cancer 2018; 103: 267–273. 30196989 10.1016/j.ejca.2018.07.135 PMC6202675

[5] Moss S Mathews C Day TJ , et al. Increased uptake and improved outcomes of bowel cancer screening with a faecal immunochemical test: results from a pilot study within the national screening programme in England. Gut 2017; 66: 1631–1644. 27267903 10.1136/gutjnl-2015-310691

[6] Palmer CK Thomas MC McGregor LM , et al. Regional alcohol consumption and alcohol-related mortality in Great Britain: novel insights using retail sales data. BMC Public Health 2015; 15: –7. 10.1186/1471-2458-15-1 PMC4324675 25563658

[7] Duffy SW Myles JP Maroni R , et al. Rapid review of evaluation of interventions to improve participation in cancer screening services. J Med Screen 2017; 24: 127–145. 27754937 10.1177/0969141316664757 PMC5542134

[8] Shankleman J Massat NJ Khagram L , et al. Evaluation of a service intervention to improve awareness and uptake of bowel cancer screening in ethnically-diverse areas. Br J Cancer 2014; 111: 1440–1447. 24983374 10.1038/bjc.2014.363 PMC4183836

[9] Cancer Research UK. Evidence on increasing bowel screening uptake. Bowel screening case studies. *Call for a Kit Clinic*, https://www.cancerresearchuk.org/health-professional/screening/bowel-screening-evidence-and-resources/evidence-on-increasing-bowel-screening-uptake#bowelcase0 (2020, accessed 18. June 2021).

[10] GOV.UK. English indices of deprivation 2019, https://www.gov.uk/government/statistics/english-indices-of-deprivation-2019 (2019, accessed 30. March 2021).

[11] Freeman HP . The origin, evolution, and principles of patient navigation. Cancer Epidemiol Biomarkers Prev 2012; 21: 1614–1617. 23045534 10.1158/1055-9965.EPI-12-0982

[12] Lim J Broughan J Crowley D , et al. COVID-19’s impact on primary care and related mitigation strategies: a scoping review. European J Gen Pract 2021; 27: 166–175. 34282695 10.1080/13814788.2021.1946681 PMC8293960

[13] Helsper CW Campbell C Emery J , et al. Cancer has not gone away: a primary care perspective to support a balanced approach for timely cancer diagnosis during COVID-19. Eur J Cancer Care 2020; 29: e13290. 10.1111/ecc.13290 PMC7361158 32633887

