# Supplementary material for: Evaluation of the Call for a Kit intervention to increase bowel cancer screening uptake in Lancashire, England
Source: J Med Screen. 2022 Apr 12;29(3):166–71. doi: 10.1177/09691413221089184 (PMC9381688; doi:10.1177/09691413221089184)
Supplement: sj-docx-1-msc-10.1177_09691413221089184 - Supplemental material for Evaluation of the Call for a Kit intervention to increase bowel cancer screening uptake in Lancashire, England [file sj-docx-1-msc-10.1177_09691413221089184.docx]

# Supplementary Tables

Table S1: Description of the calls made (N=10,772)

|  | Accepted offer for meeting or phone consultation (N=3,529) | |  | Did not answer or rejected offer for meeting or phone consultation (N=7,243) | |  | Total (N=10,772) | |
| --- | --- | --- | --- | --- | --- | --- | --- | --- |
|  | N | (%) |  | N | (%) |  | N | (%) |
| Gender |  |  |  |  |  |  |  |  |
| Female | 1,469 | (31.6%) |  | 3,174 | (68.4%) |  | 4,643 | (43.1%) |
| Male | 2,060 | (33.6%) |  | 4,069 | (66.4%) |  | 6,129 | (56.9%) |
| Ethnicity |  |  |  |  |  |  |  |  |
| White | 3,218 | (32.1%) |  | 6,817 | (67.9%) |  | 10,035 | (93.2%) |
| Indian | 123 | (33.5%) |  | 244 | (66.5%) |  | 367 | (3.4%) |
| Pakistani | 133 | (47.3%) |  | 148 | (52.7%) |  | 281 | (2.6%) |
| Black, mixed and others | 55 | (61.8%) |  | 34 | (38.2%) |  | 89 | (0.8%) |
| Area level deprivation |  |  |  |  |  |  |  |  |
| IMD quintile 5 [3-1,529] | 797 | (33.2%) |  | 1,600 | (66.8%) |  | 2,397 | (22.3%) |
| IMD quintile 4 [1,542-3,369] | 896 | (37.1%) |  | 1,518 | (62.9%) |  | 2,414 | (22.4%) |
| IMD quintile 3 [3,396-6,415] | 590 | (31.5%) |  | 1,287 | (68.8%) |  | 1,871 | (17.4%) |
| IMD quintile 2 [6,524-11,645] | 479 | (33.9%) |  | 932 | (66.1%) |  | 1,411 | (13.1%) |
| IMD quintile 1 [11,685-26,146] | 767 | (28.7%) |  | 1,906 | (71.3%) |  | 2,673 | (24.8%) |

Note: IMD quintiles are calculated based on the rank of the GP practice which range from 1 (the most deprived area) to 32,844 (the least deprived area)

Table S2. Difference in demographics between those who accepted the face to face meeting and those who chose the phone consultation (N=3,529)

|  | Preferred the face to face meeting (N=2,464) | |  | Preferred a phone consultation (N=1,065) | |  | p-value* |
| --- | --- | --- | --- | --- | --- | --- | --- |
|  | N | (%) |  | N | (%) |  |  |
| Gender |  |  |  |  |  |  |  |
| Female | 972 | (66.2%) |  | 497 | (33.8%) |  | <0.001 |
| Male | 1,492 | (72.4%) |  | 568 | (27.6%) |  |  |
| Age |  |  |  |  |  |  |  |
| 60-64 years | 1126 | (66.8%) |  | 560 | (33.2%) |  |  |
| 65-69 years | 913 | (72.7%) |  | 343 | (27.3%) |  | <0.001 |
| 70+ years | 425 | (72.4%) |  | 162 | (27.6%) |  |  |
| Ethnicity |  |  |  |  |  |  |  |
| White | 2,203 | (68.5%) |  | 1,015 | (31.5%) |  | <0.001 |
| Indian | 101 | (82.1%) |  | 22 | (17.9%) |  |  |
| Pakistani | 114 | (85.7%) |  | 19 | (14.3%) |  |  |
| Black, mixed and others | 46 | (83.6%) |  | 9 | (16.4%) |  |  |
| Area level deprivation |  |  |  |  |  |  |  |
| IMD quintile 1 (least deprived) | 525 | (68.4%) |  | 242 | (31.6%) |  | 0.197 |
| IMD quintile 2 | 340 | (71.0%) |  | 139 | (29.0%) |  |  |
| IMD quintile 3 | 407 | (69.0%) |  | 183 | (31.0%) |  |  |
| IMD quintile 4 | 651 | (72.7%) |  | 245 | (27.3%) |  |  |
| IMD quintile 5 (most deprived) | 541 | (67.9%) |  | 256 | (32.1%) |  |  |

*p-value refers to Chi-Square test for independence

Table S3. Description of the test kit returned (N=10,772)

|  | Returned the test kit (N=1,651) | |  | Did not answer call, did not order test kit or did not return test kit (N=9,121) | |  | Total (N=10,772) | |
| --- | --- | --- | --- | --- | --- | --- | --- | --- |
|  | N | (%) |  | N | (%) |  | N | (%) |
| Gender |  |  |  |  |  |  |  |  |
| Male | 982 | (59.5) |  | 5,147 | (56.4) |  | 6,129 | (56.9) |
| Female | 669 | (40.5) |  | 3,974 | (43.6) |  | 4,643 | (43.1 |
| Ethnicity |  |  |  |  |  |  |  |  |
| White | 1,510 | (91.5) |  | 8,525 | (93.5) |  | 10,035 | (93.2) |
| Indian | 58 | (3.5) |  | 309 | (3.4) |  | 367 | (3.4) |
| Pakistani | 53 | (3.2) |  | 228 | (2.5) |  | 281 | (2.6) |
| Black, mixed and others | 30 | (1.8) |  | 59 | (0.6) |  | 89 | (0.8) |
| Area level deprivation |  |  |  |  |  |  |  |  |
| IMD quintile 1 [11,685-26,146] | 419 | (25.4) |  | 2,254 | (24.7) |  | 2,673 | (24.8) |
| IMD quintile 2 [6,524-11,645] | 430 | (26.0) |  | 987 | (10.8) |  | 1,417 | (13.2) |
| IMD quintile 3 [3,396-6,415] | 264 | (16.0) |  | 1,607 | (17.6) |  | 1,871 | (17.4) |
| IMD quintile 4 [1,542-3,369] | 225 | (13.6) |  | 2,189 | (24.0) |  | 2,414 | (22.4) |
| IMD quintile 5 [3-1,529] | 313 | (19.0) |  | 2,084 | (22.8) |  | 2,397 | (22.3) |

* *p*<0.05; ** *p*<0.01

CI: confidence interval; IMD: Indices of multiple deprivation; OR: odds ratio.

Note: IMD quintiles are calculated based on the rank of the GP practice which range from 1 (the most deprived area) to 32,844 (the least deprived area)

Table S4: Returning the test kit (per protocol analysis, N=3,008)

|  |  | Returning the test kit | | | | | | |
| --- | --- | --- | --- | --- | --- | --- | --- | --- |
|  |  |  |  | Unadjusted model | |  | Adjusted model | |
|  | N | N | (%) | OR | 95% CI |  | OR | 95% CI |
| Overall | 3,008 | 1,651 | (54.9) |  |  |  |  |  |
| Gender |  |  |  |  |  |  |  |  |
| Female | 1,182 | 669 | (56.6) | Ref. |  |  | Ref. |  |
| Male | 1,826 | 982 | (53.8) | 0.892 | 0.770 – 1.034 |  | 0.909 | 0.783 – 1.056 |
| Ethnicity |  |  |  |  |  |  |  |  |
| White | 2,743 | 1,510 | (55.0) | Ref. |  |  | Ref. |  |
| Indian | 120 | 58 | (48.3) | 0.764 | 0.530 - 1.101 |  | 0.756 | 0.519 - 1.099 |
| Pakistani | 99 | 53 | (53.5) | 0.941 | 0.629 - 1.406 |  | 1.009 | 0.670 - 1.518 |
| Black, mixed and others | 46 | 30 | (65.2) | 1.531 | 0.831 - 2.822 |  | 1.358 | 0.729 - 2.530 |
| Area level deprivation |  |  | |  |  |  |  |  |
| IMD quintile 5 [3-1,529] | 662 | 313 | (47.3) | Ref. |  |  | Ref. |  |
| IMD quintile 4 [1,542-3,369] | 423 | 225 | (53.2) | 1.267 | 0.992 - 1.618 |  | 1.276 | 0.997 – 1.633 |
| IMD quintile 3 [3,396-6,415] | 502 | 264 | (52.6) | 1.237 | 0.980 – 1.560 |  | 1.241 | 0.982 – 1.566 |
| IMD quintile 2 [6,524-11,645] | 742 | 430 | (58.0) | 1.537 | 1.244 – 1.898** |  | 1.555 | 1.254 – 1.928** |
| IMD quintile 1 [11,685-26,146] | 679 | 419 | (61.8) | 1.797 | 1.446 – 2.233** |  | 1.790 | 1.440 – 2.225** |

* *p*<0.05; ** *p*<0.01

CI: confidence interval; IMD: Indices of multiple deprivation; OR: odds ratio.

Note: IMD quintiles are calculated based on the rank of the GP practice which range from 1 (the most deprived area) to 32,844 (the least deprived area)
